# Prediction of Water Resistance of Magnesium Oxychloride Cement Concrete Based upon Hybrid-BP Neural Network

**DOI:** 10.3390/ma16093371

**Published:** 2023-04-25

**Authors:** Penghui Wang, Hongxia Qiao, Cuizhen Xue, Qiong Feng

**Affiliations:** 1 College of Civil and Transportation Engineering, Guangdong Provincial Key Laboratory of Durability for Marine Civil Engineering, Shenzhen University, Shenzhen 518060, China; wangpenghui0327@163.com; 2 School of Civil Engineering, Lanzhou University of Technology, Lanzhou 730050, China; xuecuizhen2008@163.com (C.X.); fengqiong.1985@163.com (Q.F.)

**Keywords:** magnesium oxychloride cement concrete, water resistance, compressive strength, softening coefficient, PSO-BPNN

## Abstract

To obtain the magnesium oxychloride cement concrete (MOCC) ratio with excellent water resistance quickly and accurately, a BP neural network (BPNN) model with a topology structure of 4-10-2 was designed, and the PSO (particle swarm optimization), GWO (gray wolf optimization), and WOA (whale optimization algorithm) algorithms were used to optimize the model. The input layer parameters of the model above were n(MgO/MgCl_2_), Grade I fly ash, phosphoric acid (PA), and phosphate fertilizer (PF) content, and the output layer was the MOCC’s compressive strength and softening coefficient. The model had a dataset of 144 groups, including 100 training set data, 22 verification set data, and 22 test set data. The results showed that the PSO-BPNN model had the highest predictive accuracy among the four models, with a mean R^2^ of 0.99, mean absolute error(MAE) of 0.52, mean absolute percentage error(MAPE) of 0.01, and root mean square error (RMSE) of 0.73 in predicting compressive strength, and a mean R^2^ of 0.99, MAE of 0.44, MAPE of 0.01, and RMSE of 0.62 in predicting the softening coefficient. The results showed that using the PSO-BPNN to predict the compressive strength and softening coefficient of MOCC is feasible and can provide theoretical guidance for designing the MOCC mix.

## 1. Introduction

Magnesium oxychloride cement concrete (MOCC) is a magnesium-based cementitious material that is characterized by its light weight, high strength, and high-temperature resistance. It is also highly resistant to salt corrosion and has good durability even in environments with high salt content. Further, MOCC is a green building material that has low carbon emissions during the manufacturing process, which reduces environmental pollution [1,2]. However, its hydration products, 5Mg(OH)_2_·MgCl_2_·8H_2_O (referred to as phase 5) and 3Mg(OH)_2·_MgCl_2_·8H_2_O (phase 3), are prone to hydrolysis in moist environments, and generate Mg(OH)_2_ and MgCl_2_, which leads to a loose cement structure and increased porosity that decrease its strength [3,4]. At the same time, it also creates conditions that allow CO_2_ to enter the material, where CO_2_ reacts with Mg(OH)_2_ to produce magnesium chlorocarbonate (2MgCO_3_·Mg(OH)_2_·MgCl_2_·6H_2_O) that causes MOCC to warp and deform [5,6]. Therefore, MOCC’s poor water resistance limits its widespread use and is currently an urgent problem that needs to be solved. In the past, the primary method used to improve water resistance was to add phosphoric acid (PA) [6,7], fly ash [3], and another composite modifier [4,8] to MOCC. However, the relation between the water-resistant agent and the MOCC’s compressive strength and softening coefficient is complex and nonlinear, and there is no general equation that can reflect and explain its relevance accurately. Therefore, determining the appropriate amount of water-resistant agent to add requires significant manpower, material resources, and time [9,10], and thus, it is necessary to establish a concise general equation to reflect the nonlinear relation between the water-resistant agent and the MOCC’s compressive strength and softening coefficient, which can provide theoretical guidance to determine the dosage and type of water-resistant agent in MOCC.

With the continuous development of science and technology, artificial intelligence technology [11,12] has gradually begun to be used widely in civil engineering, for example, to predict concrete’s compressive strength based upon the composition of concrete materials, mixing temperature, and mixing time. Prediction of the shear capacity of RC beams strengthened with inorganic composites [13] and to predictthe compressive and flexural strengths of eco-friendly concrete containing recycled concrete aggregate [14]. In addition, recent studies have developed a flexural capacity prediction model as well as an efficient and user-friendly software tool for both fiber-reinforced polymer (FRP)-RC beams [15] and corroded RC beams [16]. The process of establishing the above model is achieved through training and evaluating various ML models, ranging from the simplest to the most complex, to determine the most efficient and accurate model [17]. However, the above model cannot achieve multioutput prediction. Among various artificial intelligence algorithms, BPNN [18,19] has been applied widely to predict the strength of rubber concrete, recycled concrete, and the bond strength of reinforcement because of its self-organization, self-adaptation, and ability to solve complex nonlinear mapping problems. However, the BPNN algorithm has a slow convergence speed and is prone to fall into local optimal solutions [20,21,22] in the process of seeking the optimal solution, which results in low predictive accuracy, particularly when multiple input and output factors are involved. Therefore, it is necessary to use optimization algorithms to improve the BP neural network (BPNN) algorithm, so that it can achieve rapid convergence speed and global search ability, and predict MOCC’s water resistance accurately.

Inspired by various animal group behaviors in nature, experts and scholars have proposed swarm intelligence optimization algorithms to optimize the BPNN algorithm and reach the goal of achieving global optimal solutions that can be applied to solve large-scale, high-dimensional problems based upon different activity mechanisms within animal populations. Algorithms that are used commonly include the gray wolf optimization algorithm (GWO) [23], whale optimization algorithm (WOA) [24], particle swarm optimization algorithm (PSO) [25], etc. These algorithms take the population particles as the starting position of the target function to be processed and simulate the movement update like an animal group to identify the best position and find a suitable global optimal solution ultimately. Currently, some optimized BPNNs, such as the GWO-BPNN, WOA-BPNN, and PSO-BPNN, have been used to predict the compressive strength of concrete [20,22,25]. However, there is no relevant research on predicting the concrete mix ratio design with multiple parameters and double outputs. In summary, the optimized BPNN has good predictive ability in the physical and service performance of concrete, making it possible to predict the relation between MOCC raw materials and water resistance performance.

Therefore, in view of the excellent prediction ability of the optimized BPNN and the gap in the design of the MOCC water resistance mix ratio, this paper takes the influence of MOCC raw materials on its water resistance as the research object, and analyzes the water resistance effect through long-term water resistance tests. GWO, WOA, and PSO algorithms were used to establish the neural network prediction model of MOCC water resistance, which provide guidance for the prediction of MOCC water resistance and provide a scientific basis for the design of the MOCC mix ratio.

## 2. Test Method

### 2.1. Raw Materials

The raw materials in MOCC are calcined magnesium oxide (MgO), magnesium chloride (MgCl_2_), water-reducing agent, water-resistant agent, fly ash, and coarse and fine aggregates. The chemical composition of the cementitious material, MgO and MgCl_2_, is shown in Table 1 and Table 2, and the chemical composition of Class I fly ash is shown in Table 3. The fine aggregate was well-graded medium sand, which meets the requirements of the JGJ52-2006 [26]. The coarse aggregate is a continuous grading with a particle size of 5–25 mm that meets the requirements of the JGJ52-2006 as well. The water-resistant agents are PA and PF, in which the content of H_3_PO_4_ is not less than 85.0%, and the color is not more than 25 Heizeng. This meets the requirements of GB8076-2008 “Concrete Admixtures”. The main component of PF is superphosphate, and the effective phosphorus pentoxide content is more than or equal to 12%.

### 2.2. Test Plan

To prepare the MOCC specimens, the amount of MgCl_2_ solution required was prepared first according to Table 4 (in which the amounts of MgO, coarse aggregate, fine aggregate, and water-reducing agent were 389, 1162, 625, and 16.02 kg/m^3^, respectively).

Then, the amounts of sand, stones, fly ash, and MgO required were weighed according to Table 4 and mixed evenly. Next, the specimens were prepared according to the requirements of the GB/T 50082-2009 [27]. After curing for 28 d, the compressive strength test was performed. Then, the specimens were divided into two groups: Group A continued to be cured in dry conditions, and Group B was subjected to saturation testing, where the water level exceeded the specimens’ surface by 3 cm. After curing for 360 d, the compressive strength test was performed, and the softening coefficient was calculated according to Formula (1) [10].

(1) $$k=\frac{f_{w}}{f}$$

in which *f*_w_ is the MOCC’s compressive strength in the saturated environment, and *f* is its compressive strength in the dry environment.

## 3. Methodology

### 3.1. Data Description

#### 3.1.1. Data Distribution

**Figure 1:**
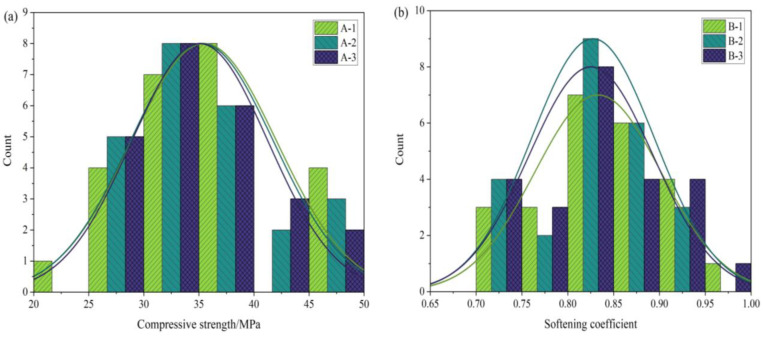
28 d physical properties of MOCC: (**a**) compressive strength; (**b**) softening coefficient.

**Figure 2:**
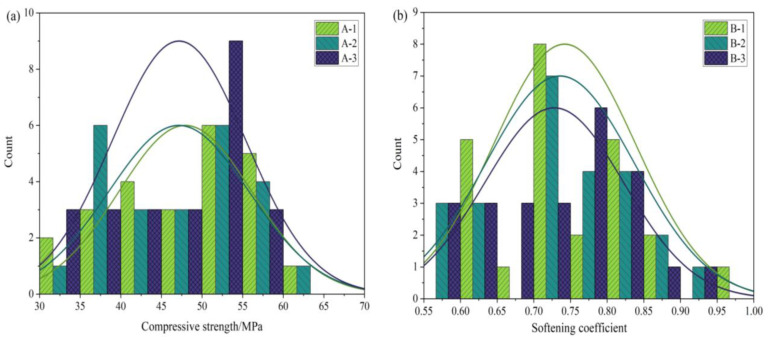
360 d physical properties of MOCC: (**a**) compressive strength; (**b**) softening coefficient.

MOCC’s compressive strength at different ages in Table 4 was obtained with the compressive strength test, and the results are shown in Figure 1 and Figure 2, in which CS-DE means compressive strength in the dry environment. As Figure 1a indicates, the MOCC’s compressive strength in the dry environment decreased at 28 d as the n(MgO/MgCl_2_) increased. The reason for this is that the concentration of MgCl_2_ affects the rate at which the MOCC’s hydration products form 3Mg(OH)_2_·MgCl_2_·8H_2_O and 5Mg(OH)_2_·MgCl_2_·8H_2_O (phase 5), in which phases 3 and 5 are porous polycrystal stacking structures with interlaced crystals, which can improve the MOCC’s strength in the dry environment. It can be seen from Figure 1b that fly ash, PA, and PF improved the MOCC’s water resistance to a certain extent.

A long-term (360 d) water resistance test was conducted to analyze different materials’ effect on the improvement of the MOCC’s long-term water resistance. The results are shown in Figure 2. From Figure 2a, it can be seen that with the continuous increase in the number of days of hydration, the MOCC’s compressive strength in the dry environment improved significantly. At 360 d, its maximum compressive strength was 64.60 MPa, which is approximately 31.3% higher than that at 28 d. From Figure 2b, in a saturated environment, the softening coefficient of MOCC with different mix ratio was 0.63–0.89, which decreased by 0.8–24% compared to 28 d. It can be seen that although fly ash, PA, and PF improved the MOCC’s water resistance, their long-term effects on water resistance still need to be improved further.

#### 3.1.2. Correlation Analysis

**Figure 3:**
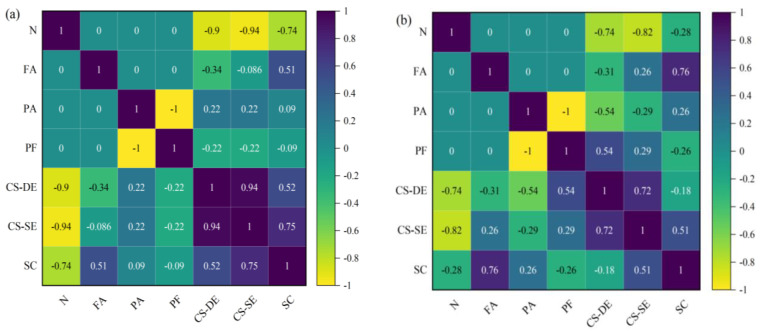
Correlation coefficient matrix: (**a**) 28 d; (**b**) 360 d.

The effect of n(MgO/MgCl_2_), Class I fly ash, PA, and PF on the MOCC’s water resistance was analyzed using the compressive strength and softening coefficient as indicators. The correlation coefficient matrix was used to analyze the correlation at different ages, and the results are shown in Figure 3, in which the darker the color in the image, the higher the correlation [28]. Figure 3a shows the correlation results at 28 d, and Figure 3b shows the results at 360 d. CS-DE represents the compressive strength in a dry environment, CS-SE represents the compressive strength in a wet environment, SC represents the softening coefficient, N represents n(MgO/MgCl_2_), FA represents Class I fly ash, PA represents phosphoric acid, and PF represents phosphate fertilizer.

From Figure 3a to Figure 3b, it can be seen that at 28 d, the correlation coefficients between n(MgO/MgCl_2_) and the MOCC’s compressive strength and softening coefficient were −0.9, −0.94, and −0.74, respectively. At 360 d, the correlation coefficients between n(MgO/MgCl_2_) and the compressive strength and softening coefficient were −0.74, −0.80, and −0.20, respectively. This indicated that the n(MgO/MgCl_2_) content was correlated negatively with the compressive strength and softening coefficient. At 28 d, the correlation coefficients between Class I fly ash and the compressive strength and softening coefficient were −0.34, −0.09, and 0.51, respectively, while at 360 d, the correlation coefficients between the three were −0.31, 0.31, and 0.51, respectively. This shows that the amount of Class I fly ash was correlated negatively with the MOCC’s compressive strength but positively with its improved water resistance.

At 28 d, the correlation coefficients between PA and the compressive strength and softening coefficient were 0.22, 0.22, and 0.09, respectively, while the correlation coefficients between PA and the compressive strength and softening coefficient at 360 d were −0.54, −0.29, and 0.26, respectively. This indicates that the amount of PA is correlated negatively with the MOCC’s long-term compressive strength but positively with its improved water resistance. At 28 d, the correlation coefficients between PF and the compressive strength and softening coefficient were −0.22, −0.22, and −0.09, respectively. At 360 d, the correlation coefficients between the three were 0.54, 0.29, and −0.26, respectively. This indicates that the amount of PF is correlated positively with the MOCC’s compressive strength, while its correlation with improved water resistance was both positive and negative. This research result is consistent with the research results of Gong [2] and Deng [7].

### 3.2. Procedure for Developing the Prediction Model

**Figure 4:**
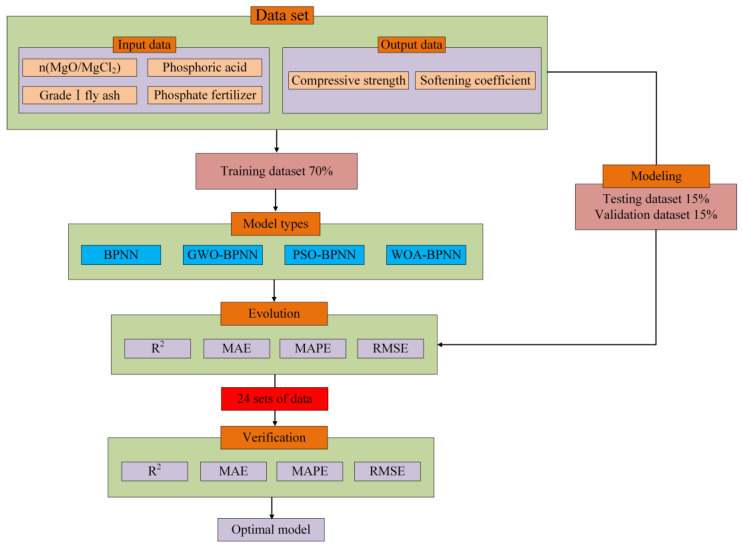
Flowchart of establishing the model.

Figure 4 shows the flowchart of establishing the prediction model of MOCC’s compressive strength and softening coefficient, which includes several steps:

Step 1: Determine the dataset. Four factors that affect MOCC’s water resistance, n(MgO/MgCl_2_), Class I fly ash, PA, and PF, were selected as input parameters, and the MOCC’s compressive strength and softening coefficient were selected as output parameters. All data were derived from the 144 datasets in this experiment. A total of 100 sets of data (70%) were used to train the neural network model, 22 (15%) were used for validation (validation dataset), and 22 (15%) were used for testing (testing dataset).

Step 2: Model hyperparameter determination. According to the relevant literature, determine the number of hidden layers in the neural network, the number of hidden nodes, the model learning rate, population size and layout, and other parameters

Step 3: Train the model. Based upon the established dataset, BPNN, PSO-BPNN, GWO-BPNN, and WOA-BPNN were trained.

Step 4: Evaluate the model. The statistical parameters RMSE, MAE, MAPE, and R^2^ were used to evaluate the four models’ predictive ability.

Step 5: Validate the model. A total of 24 sets of measured data were used to validate the established Hybrid-BPNN and determine the best neural network optimization algorithm.

### 3.3. Theoretical Background

#### 3.3.1. BPNN

The design of the BP network mainly includes the determination of the number of network layers, input layer nodes, hidden layer nodes, output layer nodes, and transfer function. The BP network can contain one or more hidden layers, but it has been proved theoretically that a network with a single hidden layer can achieve any nonlinear mapping by appropriately increasing the number of neuron nodes. Therefore, the network structure in this paper is set to one hidden layer. The input layer nodes are n(MgO/MgCl_2_), Grade I fly ash, phosphoric acid (PA), and phosphate fertilizer (PF) content that affect the water resistance of MOCC. The number of hidden layer nodes is firstly selected according to the empirical Formula (2) in this study. The output layer was the MOCC’s compressive strength and softening coefficient. The BP neural network generally uses the Sigmoid function or linear function as the transfer function. According to whether the output value contains negative values, the Sigmoid function can be divided into the Log-Sigmoid function and the Tan-Sigmoid function; the Log-Sigmoid function is characterized in that it can map data in the range (−∞,+∞) to the interval (0,1), while the output of the Tan-Sigmoid function will be limited to (−1,1). MOCC‘s compressive strength and softening coefficient cannot appear negative, so choose the Log-Sigmoid function as the transfer function of this model. The transfer function is shown in Formula (3) [29].

(2) $$m=k\;\log_{2}^{n}$$

(3) $$f(x)=\frac{1}{1+e^{-x}}$$

in which, *m* is the number of hidden layer nodes, *n* is the number of input layer nodes, and *k* is the correction coefficient for calculating hidden layer nodes.

**Figure 5:**
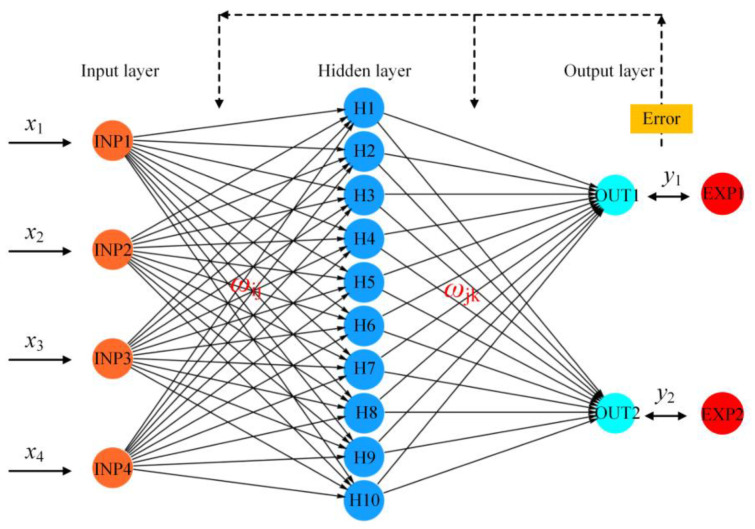
Structure diagram of BPNN.

The core idea of BPNN is the error gradient descent method. In selecting samples for training, it adjusts its weight and threshold continuously, so that the error function decreases along the negative gradient direction, making the actual output infinitely close to the expected output. BPNN can approach any continuous function continuously, its own nonlinear mapping ability is very strong, and the parameters, such as the number of hidden layers in the network, the number of processing units in each layer, and the network’s learning coefficient, can be changed flexibly according to the actual needs, with strong adaptability [19]. The structure diagram of BPNN is shown in Figure 5 [29].

In Figure 5, *x*_n_ is the input variable and *i*, *j*, and *k* are the number of nodes in the input layer, hidden layer, and output layer, respectively, *w*_ij_ and *w*_jk_ are the connection weights between the layers, and *y*_ij_ and *y*_jk_ are the output values of the hidden layer and the output layer.

#### 3.3.2. Particle Swarm Optimization Algorithm

**Figure 6:**
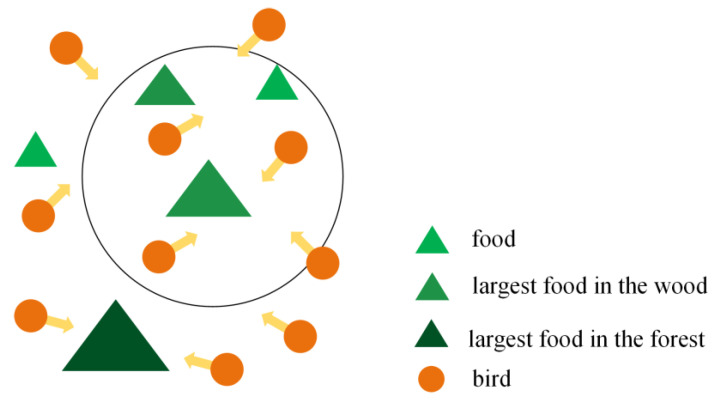
The foraging schematic diagram of PSO.

The core idea of Particle Swarm Optimization (PSO) is to obtain the optimal solution by simulating the information shared among individuals when birds are foraging. The foraging schematic diagram is shown in Figure 6 [25]. First, all particles are given random initial positions and velocities, and then the best-known global positions in the problem space are approximated according to the particles’ best-known positions, and the global optimal solution is obtained through continuous iteration. In the solution process, each particle’s speed and position are determined by its own best past position and the best past position of the entire group or the nearest neighbor, which can be expressed by Formula (4) [25].

(4) $$\left\{ \begin{array}{l} v_{ij}\left(t+1\right)=\omega v_{ij}\left(t\right)+r_{1}c_{1}\left(p_{ij}\left(t\right)-x_{ij}\left(t\right)\right)+r_{2}c_{2}\left(g_{ij}\left(t\right)-x_{ij}\left(t\right)\right)\\ x_{ij}\left(t+1\right)=x_{ij}\left(t\right)+v_{ij}\left(t+1\right) \end{array}\right.$$

in which $v_{ij}\left(t\right)$ is the velocity vector of the particle at time *t*, $x_{ij}\left(t\right)$ is the position vector of the particle at time *t*, $v_{ij}\left(t+1\right)$ is the velocity vector of the particle at time *t* + 1, $x_{ij}\left(t+1\right)$ is the position vector of the particle at time *t* + 1, $P_{ij}\left(t\right)$ is the optimal position of the individual at time *t*, $g_{ij}\left(t\right)$ is the optimal position of the group at time *t*, and ω is the best inertial weight, the value of which is in the [0,1] interval. In general applications, the adaptive value method is adopted, *c*_1_, *c*_2_ are the learning factors, and *r*_1_, *r*_2_ are two random functions with a value range of [0,1], which is used to increase the randomness of search.

#### 3.3.3. WOA-BPNN

**Figure 7:**
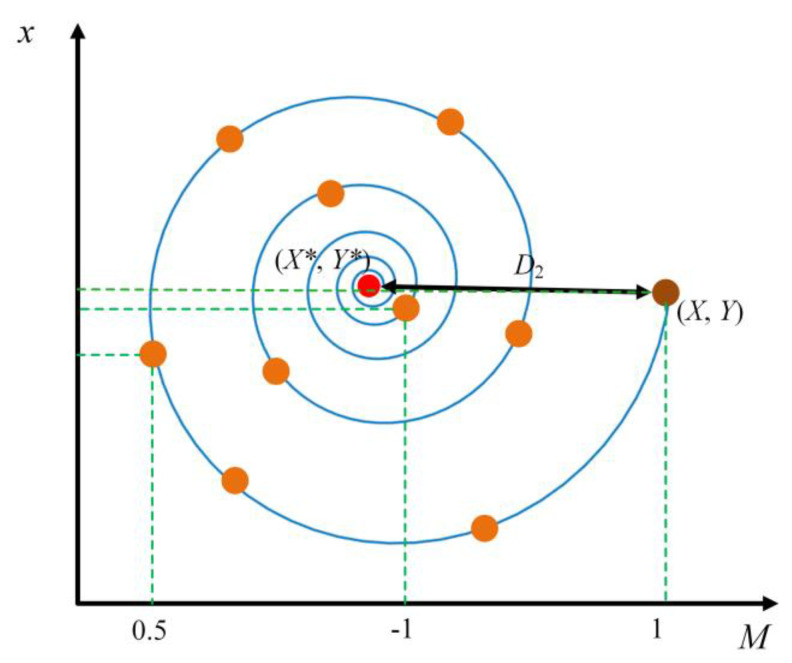
Spiral updating position.

The inspiration for the whale optimization algorithm is derived from humpback whales’ hunting strategy. The algorithm’s update mechanism includes the stages of shrinking and surrounding the prey, of bubble net attacking and hunting, and of random walk prey searching. The process has the advantages that it is simple, converges rapidly, demonstrates excellent performance in solving optimization problems, and can be applied in many fields. The bubble net attack behavior, which is the contraction and encirclement mode, and the spiral upward mode are carried out at the same time (the spiral update position diagram is shown in Figure 7 [24]). The probability of the above predatory mode is approximately 50%. The mathematical model of the bubble net attack hunting stage is defined as [24]

(5) $$X_{i}^{T+1}=\left\{ \begin{array}{ll} X_{b}^{T}-A\times D_{1} & p<0.5\\ X_{b}^{T}+D_{2}\cdot e^{ZM}\cdot \cos(2\pi M) & p\geq 0.5 \end{array}\right.$$

in which *p* ∈ [0,1], $D_{2}=\left|X_{b}^{T}-X_{i}^{T}\right|$, *Z* is the helix constant, *M* is the random number, and the value range is [−1,1]. $X_{b}^{T}$ is the position of the best individual whale when searching for the *T* generation population, i.e., the position of the target prey, and *D*_1_ is the surrounding step length. When *p* ≥ 0.5 it enters the spiral update stage; when *p* < 0.5 the algorithm enters the shrinking and enveloping stage.

#### 3.3.4. GWO-BPNN

**Figure 8:**
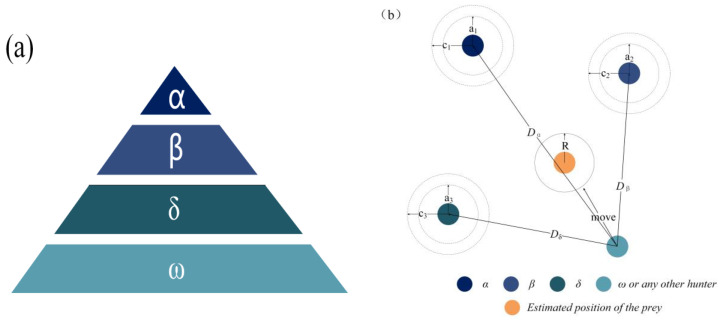
(**a**) hierarchy of grey wolf (**b**) Position updating in GWO.

The grey wolf has a strict social hierarchy, as shown in Figure 8a [23], in which the α is the leader and is responsible for all decisions; the β helps the α make decisions and assumes responsibility when necessary; δ is subject to α; β is responsible for sentry, reconnaissance, and other affairs; while ω is at the bottom of the hierarchy, and is responsible for balancing the population’s internal relationships. The hunting process includes three steps primarily: (1) Tracking and approaching prey; (2) Harassing, pursuing, and encircling prey; (3) Attacking prey. The core idea of the GWOA is to assume that α, β, and δ understand the potential location of prey better, i.e., the location of the best solution in the decision-making space of optimization problems, and other wolves update their own position basis on gray wolves α, β, and δ, and approach the prey gradually (optimal solution). The schematic diagram of the gray wolf position update is shown in Figure 8b [23]. The behavior of gray wolves during collective hunting is defined as follows [23].

(6) $${\vec{D}}_{\alpha }=\left|\vec{C_{1}\cdot }{\vec{X}}_{\alpha }-\vec{X}\right|,{\vec{\;D}}_{\beta }=\left|\vec{C_{2}\cdot }{\vec{X}}_{\beta }-\vec{X}\right|,\;{\vec{D}}_{\delta }=\left|\vec{C_{2}\cdot }{\vec{X}}_{\delta }-\vec{X}\right|$$

(7) $${\vec{X}}_{1}=\left|{\vec{X}}_{\alpha }-\vec{A_{1}}\cdot ({\vec{\;D}}_{\alpha })\right|,{\vec{X}}_{2}=\left|{\vec{X}}_{\beta }-\vec{A_{2}}\cdot ({\vec{\;D}}_{\beta })\right|,\;{\vec{X}}_{3}=\left|{\vec{X}}_{\delta }-\vec{A_{3}}\cdot ({\vec{\;D}}_{\delta })\right|$$

(8) $$\vec{X}\left(t+1\right)=\frac{\left({\vec{X}}_{1}+{\vec{X}}_{2}+{\vec{X}}_{3}\right)}{3}$$

in which $\vec{D_{\alpha }}$, $\vec{D_{\beta }}$, and $\vec{D_{\delta }}$ represent *α*, *β*, and *δ*’s distance from other individuals, respectively, and $\vec{X_{\alpha }}$, $\vec{X_{\beta }}$, and $\vec{X_{\delta }}$ represent the location of *α*, *β*, and *δ*, respectively. $\vec{X}$ is the wolf’s current location, and $\vec{X}\left(t+1\right)$ is the wolf’s updated location in the next time step. $\vec{A}$ and $\vec{C}$ add randomness to the algorithm to prevent falling into local optimization. When $\left|\vec{A}\right|<1$ the gray wolf approaches the prey and attacks it, and when $\left|\vec{A}\right|>1$ the gray wolf remains away from the prey to explore. When calculating $\vec{D}$, $\vec{C}$ will strengthen or weaken the target’s influence on $\vec{X}$ randomly.

#### 3.3.5. Evaluation Indicators

To evaluate the predictive effect of the Hybrid-BPNN model on MOCC’s softening coefficient, different models’ predictive accuracies were evaluated with the mean square error (MSE), root mean square error (RMSE), mean absolute error (MAE), and correlation coefficient (R). MAE is used to measure the absolute error between the measured value and the predicted value, MAPE is used to measure the percentage of the model’s predicted error, and RMSE is used to measure the deviation between the observed value and the true value. The expressions of the four statistical parameters are as follows [30,31].

(9) $$\mathrm{RMSE}=\sqrt{\frac{1}{m}{\sum_{i=1}^{m}\left(y_{ri}-\overset{\wedge }{y_{pi}}\right)}^{2}}$$

(10) $$\mathrm{MAE}=\frac{1}{m}\sum_{i=1}^{m}\left|\left(y_{ri}-\overset{\wedge }{y_{pi}}\right)\right|$$

(11) $$\mathrm{MAPE}=\frac{1}{m}\sum_{i=1}^{m}\left|\left(\frac{y_{ri}-\overset{\wedge }{y_{pi}}}{y_{ri}}\right)\right|\times 100$$

(12) $$R=\frac{\sum_{i=1}^{m}\left(y_{ri}-\overset{\wedge }{y_{ri}}\right)\left(y_{pi}-\overset{\wedge }{y_{pi}}\right)}{\sqrt{\sum_{i=1}^{m}{\left(y_{ri}-\overset{\wedge }{y_{ri}}\right)}^{2}\sum_{i=1}^{m}{\left(y_{pi}-\overset{\wedge }{y_{pi}}\right)}^{2}}}$$

in which $y_{ri}$ is the true compressive strength or resistivity of conductive cement mortar, and $\hat{y_{ri}}$ is the mean value that corresponds to the true value. $y_{pi}$ is the conductive cement mortar’s predicted compressive strength or resistivity, and $\hat{y_{pi}}$ is the mean value that corresponds to the predicted value. M is the total number of samples in the dataset.

#### 3.3.6. Principal Component Analysis

Principal component analysis aims to transform multivariate high-dimensional space problems into low-dimensional spaces, replacing the original variables with a few independent comprehensive variables (linear combinations of the original variables) for analysis and processing, thereby making complex problems relatively intuitive and simple.

If there are *n* samples in dataset X and each data sample contains p variables, then there are

(13) $$X=\left[\begin{array}{cccc} x_{11} & x_{12} & \cdots & x_{1p}\\ x_{21} & x_{22} & \cdots & x_{2p}\\ \vdots & \vdots & \vdots & \vdots \\ x_{n1} & x_{n2} & \cdots & x_{np} \end{array}\right]=\left[x_{1},x_{2},\cdots ,x_{p}\right]$$

in which $x_{i}={\left(x_{1i},x_{2i},\cdots x_{ni}\right)}^{T},i=1,2,\cdots p$.

Principal component analysis is the linear combination of the original *p* variables *x*_1_, *x*_2_, …, *x_p_* to form *p* new comprehensive variables, namely

(14) $$\left\{ \begin{array}{c} F_{1}=\omega_{11}x_{1}+\omega_{21}x_{2}+\cdots +\omega_{p1}p\\ F_{2}=\omega_{12}x_{1}+\omega_{22}x_{2}+\cdots +\omega_{p2}p\\ \vdots \\ F_{p}=\omega_{1p}x_{1}+\omega_{2p}x_{2}+\cdots +\omega_{pp}p \end{array}\right.$$

in which *F*i and *X*i are both n-dimensional vectors.

## 4. Results and Discussion

### 4.1. Determining the Optimal Mix Ratio

**Figure 9:**
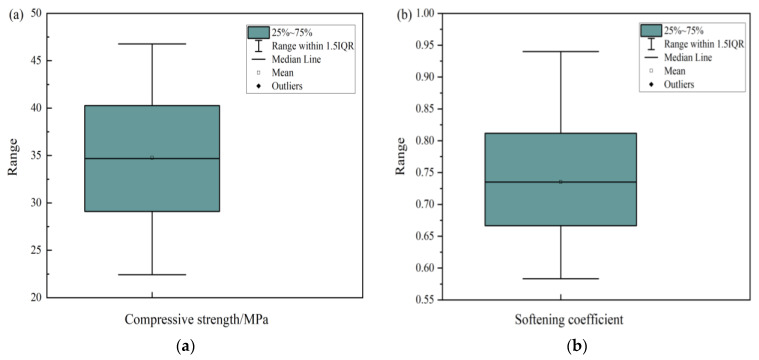
Box and Whisker plot result: (**a**) Compressive strength; (**b**) Softening coefficient.

The effects of Grade I fly ash, PA, PF, and n(MgO/MgCl_2_) on the MOCC’s improved water resistance were complex and variable. To distinguish the advantages and disadvantages of different combinations, the principal component analysis method [32] was used to study the effect of the modified materials above on the MOCC’s water resistance. PCA is sensitive to outliers in the data, which can affect the selection of principal components. Outliers can skew the variance–covariance matrix and lead to the selection of components that do not represent the underlying data structure. Therefore, a Box and Whisker plot was used to analysis the outliers; the results were shown in Figure 9. It can be seen from Figure 9 that the compressive strength and softening coefficient values are evenly distributed, and there are no outliers. The compressive strength and softening coefficient were taken as variables, and different ratios’ characteristic values and contribution rates were analyzed to determine the optimal combination among the ratios above.

**Figure 10:**
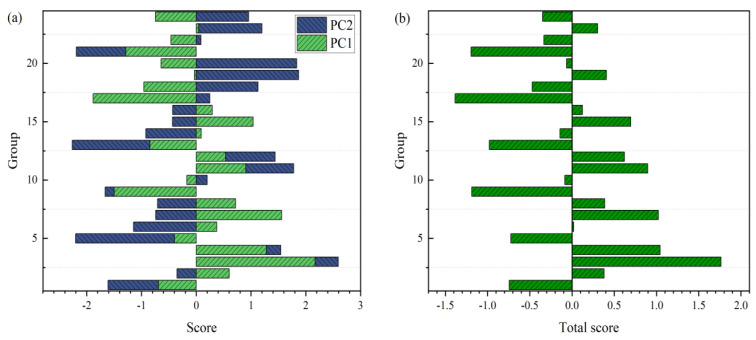
Principal component analysis result: (**a**) Principal component score; (**b**) Comprehensive score.

Principal component analysis was performed with the compressive strength and softening coefficient as variables, and the results of the principal component eigenvalue and cumulative contribution rate are shown in Table 5, while the scores of each principal component are shown in Figure 10a. Table 5 shows the selection of the number of principal components. Generally, there are two methods to extract the number of principal components: the first is to extract the principal components with a characteristic value greater than 1, and the second is to extract the principal components with a cumulative contribution rate of 85%. The second method was used in this study [33]. The principal component’s comprehensive score is equal to the sum of each principal component’s scores and its contribution rate [34]. Each component’s comprehensive score was calculated as shown in Figure 10b. From the figure, it can be concluded that the principal component’s comprehensive score is positive and negative, but this positive and negative value indicates only the comprehensive position of the performance of MOCC with different proportions and does not reflect the level of MOCC’s performance with specific proportions. A comparative analysis of the principal components’ comprehensive scores demonstrated that the comprehensive score of AMPC-1-2 was 1.76, which was the highest among all matching ratios. Therefore, AMPC-1-2 can be considered the optimal ratio, and this result is consistent with Gong’s [9] result of the optimal water tolerance ratio calculated using the Taguchi method.

### 4.2. Model Establishment

**Figure 11:**
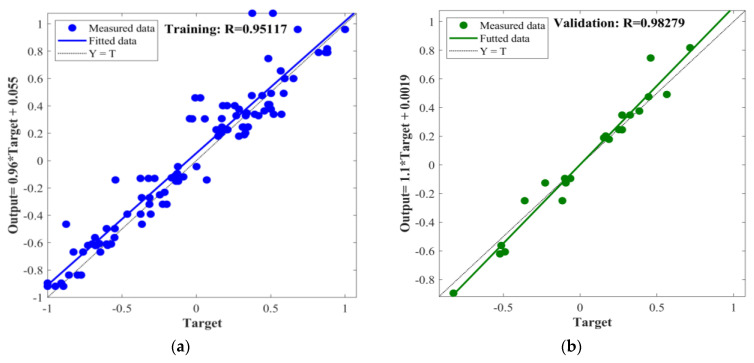

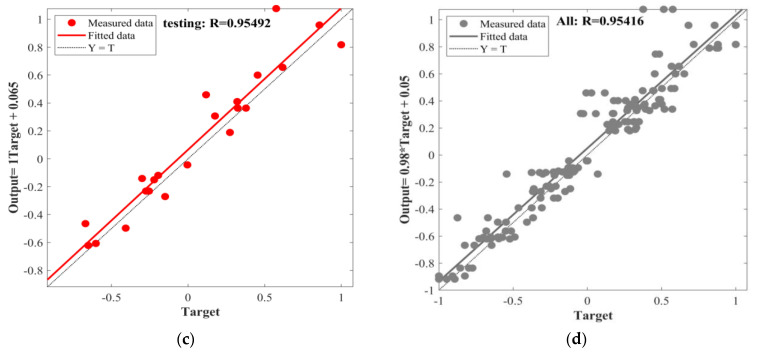
BPNN: (**a**) training; (**b**) validation; (**c**) testing; (**d**) all.

**Figure 12:**
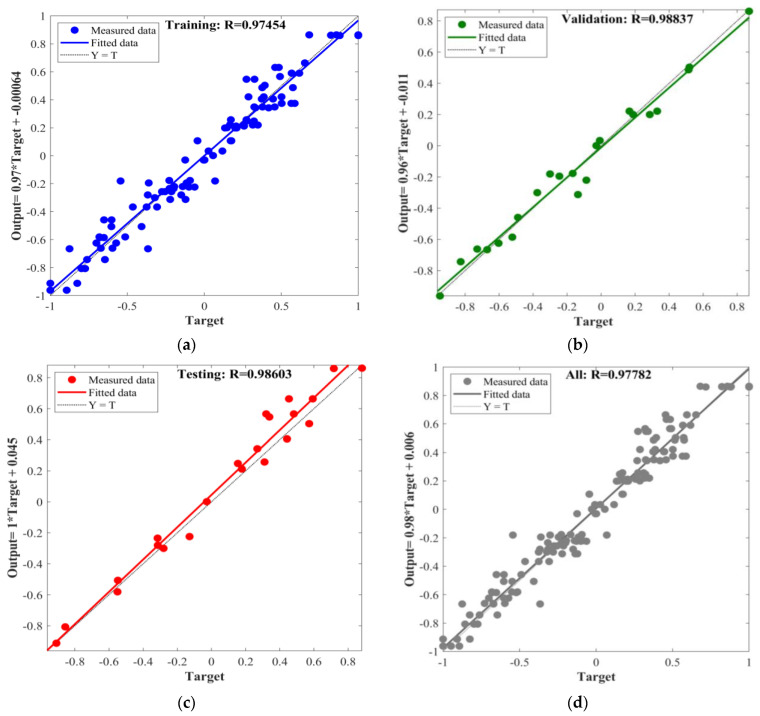
GWO-BPNN: (**a**) training; (**b**) validation; (**c**) testing; (**d**) all.

**Figure 13:**
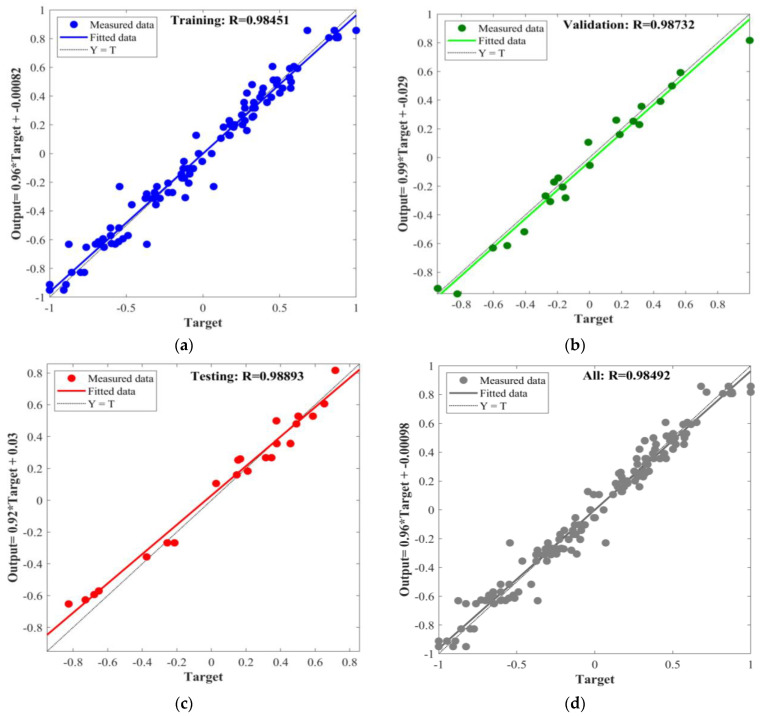
PSO-BPNN: (**a**) training; (**b**) validation; (**c**) testing; (**d**) all.

**Figure 14:**
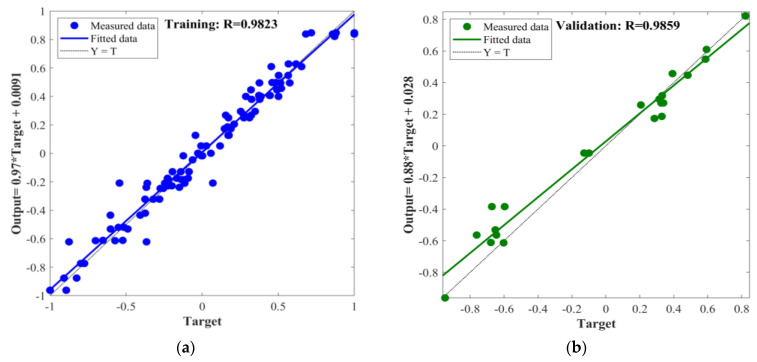

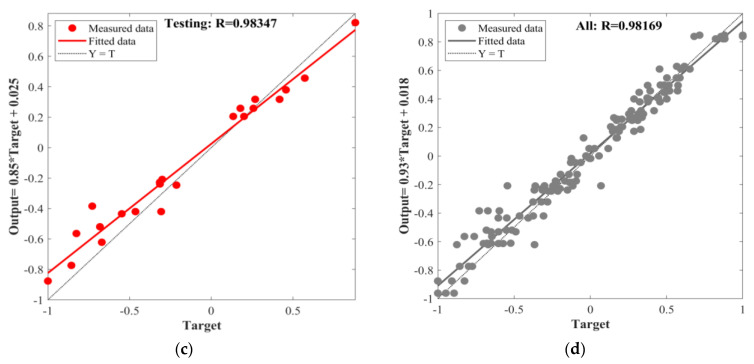
WOA-BPNN: (**a**) training; (**b**) validation; (**c**) testing; (**d**) all.

In this study, the BPNN, PSO-BPNN, GWO-BPNN, and WOA-BPNN models were used to establish predictive models for MOCC’s compressive strength and softening coefficient. To evaluate the different models’ predictive ability accurately, the correlation coefficient, R, was used to conduct a preliminary analysis of their predictive ability. In the process of establishing the models above, the total dataset consisted of 144 groups, of which 70% (100 groups) of the data were selected randomly for network training, 15% (22 groups) were selected randomly for validation, and 15% (22 groups) were selected randomly for testing. The input and output fitting curves of the network training, validation, and testing for the models are shown in Figure 11, Figure 12, Figure 13 and Figure 14.

**Figure 15:**
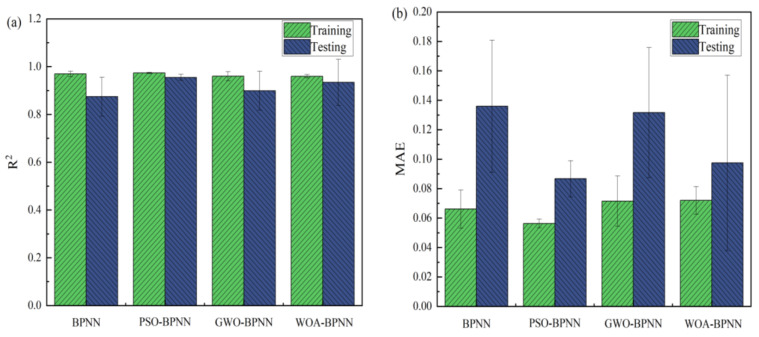

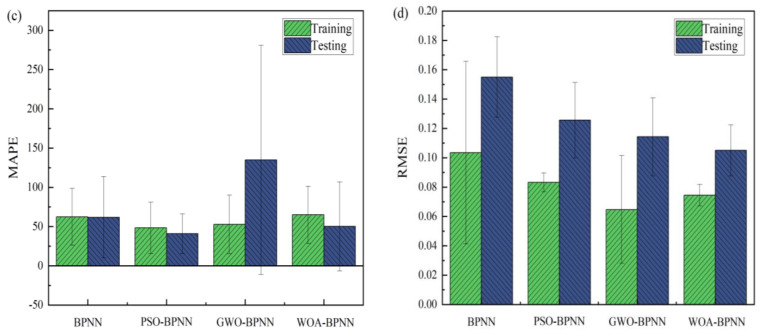
Evaluation indicators: (**a**) R^2^; (**b**) MAE; (**c**) MAPE; (**d**) RMSE.

The figures show that the correlation between the input and output values of the models above can be expressed by a linear relation. The correlation coefficients of the training process of BPNN, GWO-BPNN, PSO-BPNN, and WOA-BPNN were 0.98, 0.99, 0.99, and 0.98, respectively. The correlation coefficients of the validation process of the models were 0.94, 0.97, 0.98, and 0.10, respectively, while the correlation coefficients of the test process were 0.87, 0.96, 0.98, and 0.10, respectively. To establish the model overall, the correlation coefficients were 0.95, 0.98, 0.98, and 0.98, respectively. It can be seen that the predictive ability of BPNN optimized with the GWO, PSO, and WOA algorithms was better than that of BPNN without optimization, and the correlation coefficient overall was greater than 0.98. Then, R^2^, MAE, MAPE, and RMSE were used to evaluate the stability of the four models’ comprehensive predictive ability further. The results of the 10 predictions of the evaluation indicators above are shown in Figure 15.

As Figure 15 illustrates, the relation between the determination coefficients of the training sets of the different training models was $R_{\mathrm{PSOBPNN}}^{2}>R_{\mathrm{BPNN}}^{2}>R_{\mathrm{GWOBPNN}}^{2}>R_{\mathrm{WOABPNN}}^{2}$: The relation between the size of the average determination error was MAE_PSOBP_ < MAE_BPNN_ < MAE_GWOBP_ < MAE_WOABP_, the relation between the size of the average absolute percentage error was MAPE_PSOBP_ < MAPE_GWOBP_ < MAPE_BP_ < MAPE_WOABP_, and the relation between the size of the root mean square error was RMSE_GWOBP_ < RMSE_WOABP_ < RMSE_PSOBP_ < RMSE_BP_. The determination coefficient of the test set was $R_{\mathrm{PSOBPNN}}^{2}>R_{\mathrm{WOABPNN}}^{2}>R_{\mathrm{GWOBPNN}}^{2}>R_{\mathrm{BPNN}}^{2}$, the size relation of the average determination error was MAE_PSOBP_ < MAE_WOABPNN_ < MAE_GWOBP_ < MAE_BP_, the size relation of the average absolute percentage error was MAPE_PSOBP_ < MAPE_WOABP_ < MAPE_BP_ < MAPE_GWOBP_, and the size relation of the root mean square error was RMSE_WOABP_ < RMSE_GWOBP_ < RMSE_PSOBP_ < RMSE_BP_. Therefore, in this study, it can be concluded preliminarily that the PSO-BPNN model is the best prediction model, as it has greater advantages in predicting MOCC’s compressive strength and softening coefficient. The PSO algorithm is optimized by the probability search method, and has no control constraints on individuals. It does not affect the solution of the model’s entire problem because individual samples are outliers, which makes the model more robust; the fitness function and the agent update algorithm in the algorithm are characterized by decoupling and are suitable to expand the system [35].

### 4.3. Verification of the Model’s Predictive Ability

**Figure 16:**
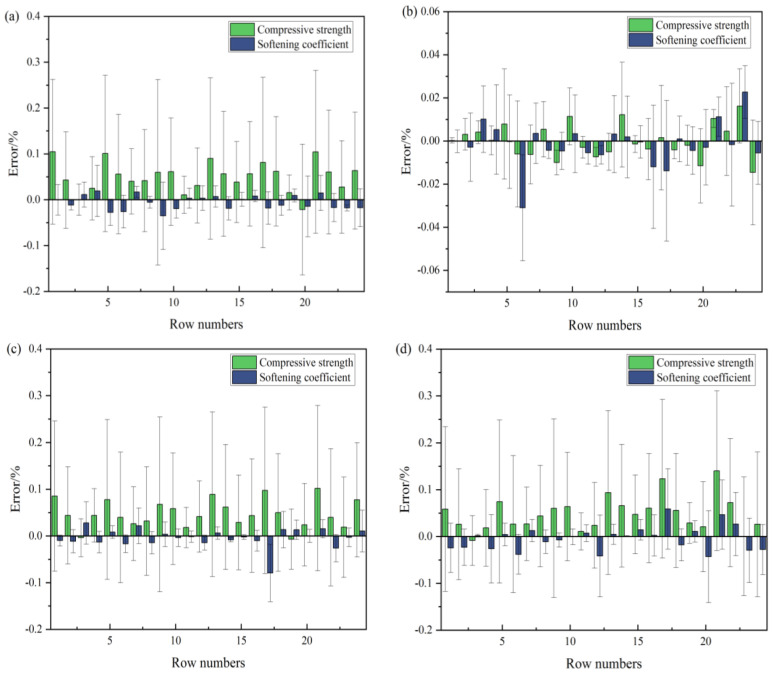
Error of different algorithms: (**a**) BPNN; (**b**) PSO-BPNN; (**c**) GWO-BPNN; (**d**) WOA-BPNN.

**Figure 17:**
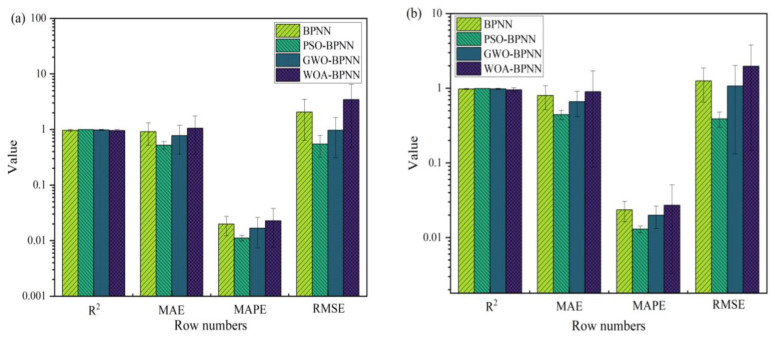
Evaluation index of model validation: (**a**) Compressive strength evaluation index; (**b**) Softening coefficient evaluation index.

Through the error analysis in the process of establishing the previous model, it was determined that the PSO-BPNN largely had the highest accuracy in predicting MOCC’s compressive strength and water resistance. To verify the model’s predictive ability, 24 * 2 groups of data were selected randomly from the dataset, and the trained model above was used for prediction. The error in the test results is shown in Figure 16, which shows the prediction error of the MOCC’s compressive strength, softening coefficient, and the actual value. It can be seen from Figure 17a that the error range in the BPNN model’s prediction of compressive strength and the softening coefficient was −0.67–0.28% and −0.05–0.08%, respectively, the error range in the PSO-BPNN model’s prediction of compressive strength and the softening coefficient was −0.04–0.03% and −0.053–0.037%, respectively, and the error range in the GWO-BPNN model’s prediction of compressive strength and the softening coefficient was −0.15–0.27% and −0.15–0.09%, respectively. Finally, the error range in the WOA-BPNN model’s prediction of compressive strength and the softening coefficient was −0.12–0.33% and −0.15–0.15%, respectively. It can be concluded that the Hybrid-BPNN model’s error is not more than ±0.5, which shows that the models above have good predictive ability.

To analyze the predictive ability of the four models in this study further, R^2^, MAE, MAPE, and RMSE were calculated. The results are shown in Figure 17, in which Figure 17a shows the compressive strength’s evaluation index and Figure 17b shows the softening coefficient’s evaluation index. From Figure 17, we can see that R^2^_PSO-BPNN_(0.98) > R^2^_GWO-BPNN_ > R^2^_BPNN_ > R^2^_WOA-BPNN_, MAE_BPNN_ > MAE_WOA-BPNN_ > MAE_GWO-BPNN_ > MAE_PSO-BPNN_, MAPE_BPNN_ > MAPE_WOA-BPNN_ > MAPE_GWO-BPNN_ > MAPE_PSO-BPNN_, and RMSE_BPNN_ > RMSE_WOA-BPNN_ > RMSE_GWO-BPNN_ > RMSE_PSO-BPNN_. According to each statistical parameter’s results, the PSO-BPNN model has the strongest predictive ability and the highest stability. This demonstrates that it can be used to predict MOCC’s long-term compressive strength and softening coefficient. This result is consistent with those of Van [28] and Han [35], who concluded in their research that the Gradient-Boosting PSO optimization model is stronger than a single machine learning model. Through verification and analysis, it was shown further that the optimized BPNN model is superior to one that is not optimized, and the PSO algorithm has the highest optimization accuracy. This is because PSO has certain advantages among evolutionary algorithms. For example, it does not have complex operators as evolutionary algorithms [25] and achieves stronger global optimization capabilities through a random search of each particle in the solution space [34]. The combination of BPNN and the PSO optimization algorithm can improve the degree of the BPNN model’s fit to the data and has higher predictive accuracy.

## 5. Conclusions

In this study, BPNN, PSO-BPNN, GWO-BPNN, and WOA-BPNN were used to establish a model to predict the long-term compressive strength and electric softening coefficient of MOCC. The models’ input parameters were n(MgO/MgCl_2_); the content of primary fly ash, PA, and PF in the hidden layer was 10; and the output parameters were compressive strength and the softening coefficient. The optimal ratio for MOCC’s long-term water resistance was obtained by principal component analysis, and the specific results were as follows:

1. The influence of water-resistant raw materials on MOCC’s compressive strength and softening coefficient was analyzed using the correlation coefficient matrix, and it was found that the content of n(MgO/MgCl_2_) was correlated negatively with the compressive strength and softening coefficient. The content of Class I fly ash and PA was correlated negatively with the compressive strength, and positively with the improvement in water resistance. The PF content was correlated positively with the compressive strength, and correlated both positively and negatively with the improvement in water resistance.
2. The comprehensive score of AMPC-1-2 was 1.76 in the comparative analysis of the principal components’ comprehensive scores, which was the highest among all of the matches. Therefore, it was concluded that AMPC-1-2 is the optimal ratio.
3. The dual factor output of MOCC’s compressive strength and softening coefficient was one of the main results of this study. The predictive ability of the PSO-BPNN, WOA-BPNN, and GWO-BPNN models was more powerful than that of the BPNN model, and among the three optimization models, the PSO-BPNN model had the highest predictive accuracy. In the prediction of compressive strength, the mean value of each evaluation parameter of PSO-BPNN was R^2^ = 0.10, MAE = 0.52, MAPE = 0.01, and RMSE = 0.73, while in the prediction of the softening coefficient, the mean value of each evaluation parameter was R^2^ = 0.10, MAE = 0.44, MAPE=0.01, and RMSE = 0.62.

Finally, this article preliminarily established a MOCC compressive strength and water resistance prediction model, but the water resistance influencing factors mentioned in the article were only four, and the established prediction model is still incomplete. In addition, this article has not fully explained the effectiveness of the prediction model. Additionally, future research can investigate the durability degradation model of MOCC under the coupling of multiple factors.

## Figures and Tables

**Table 1:** Magnesium oxide chemical composition.

MgO	MgO	CaO	SiO_2_	Loss of Ignition	Others
90	48.6	1.1	3.2	3.8	1.9

**Table 2:** Magnesium chloride chemical composition.

MgCl_2_·6H_2_O	SO_4_^−^	K^+^ + Na^+^	CaCl_2_	Other
96	0.4	1.2	0.4	2.0

**Table 3:** Grade I fly ash chemical composition (%).

Mg	Ca	Fe_2_O_3_	Al_2_O	SO	Loss of Ignition	SiO_2_
1.19	5.3	9.43	20.93	0.41	3.26	54.32

**Table 4:** MOCC Mix Ratio (kg/m^3^).

Test No.	MgCl_2_ Solution	Grade I Fly Ash	Phosphate	Phosphate Fertilizer	Test No.	MgCl_2_ Solution	Grade I Fly Ash	Phosphate	Phosphate Fertilizer
APMC1-0	301	0	4.58	0	APMC4-2	271	68.64	0	9.15
APMC1-1	301	34.32	4.58	0	APMC4-3	271	102.96	0	9.15
APMC1-2	301	68.64	4.58	0	APMC5-0	271	0	4.58	4.58
APMC1-3	301	102.96	4.58	0	APMC5-1	271	34.32	4.58	4.58
APMC2-0	301	0	0	9.15	APMC5-2	271	68.64	4.58	4.58
APMC2-1	301	34.32	0	9.15	APMC5-3	271	102.96	4.58	4.58
APMC2-2	301	68.64	0	9.15	APMC6-0	246	0	4.58	0
APMC2-3	301	102.96	0	9.15	APMC6-1	246	34.32	4.58	0
APMC3-0	271	0	4.58	0	APMC6-2	246	68.64	4.58	0
APMC3-1	271	34.32	4.58	0	APMC6-3	246	102.96	4.58	0
APMC3-2	271	68.64	4.58	0	APMC7-0	246	0	0	9.15
APMC3-3	271	102.96	4.58	0	APMC7-1	246	34.32	0	9.15
APMC4-0	271	0	0	9.15	APMC7-2	246	68.64	0	9.15
APMC4-1	271	34.32	0	9.15	APMC7-3	246	102.96	0	9.15

**Table 5:** Characteristic value and cumulative contribution rate.

Principal Component Number	Eigenvalue	Percentage of Variance (%)	Cumulative (%)
1	1.53	76.68	76.68
2	0.47	23.32	100

## Data Availability

The data presented in this study are available upon request from the corresponding author.
